# Systemic Immune Activation in HIV Infection Is Associated with Decreased MDC Responsiveness to TLR Ligand and Inability to Activate Naive CD4 T-Cells

**DOI:** 10.1371/journal.pone.0023884

**Published:** 2011-09-01

**Authors:** Nicole L. Yonkers, Benigno Rodriguez, Robert Asaad, Michael M. Lederman, Donald D. Anthony

**Affiliations:** 1 Divisions of Infectious and Rheumatic Diseases, Department of Medicine, Case Western Reserve University Center for AIDS Research, Case Medical Center and VA Medical Center, University Hospitals, Cleveland, Ohio, United States of America; 2 Divisions of Infectious and Rheumatic Diseases, Department of Pathology, Case Western Reserve University Center for AIDS Research, Case Medical Center and VA Medical Center, University Hospitals, Cleveland Ohio, United States of America; Karolinska Institutet, Sweden

## Abstract

**Background:**

HIV infection is characterized by ineffective anti-viral T-cell responses and impaired dendritic cell (DC) functions, including response to Toll-Like Receptor (TLR) ligands. Because TLR responsiveness may affect a host's response to virus, we examined TLR ligand induced Myeloid and Plasmacytoid DC (MDC and PDC) activation of naïve T-cells in HIV+ subjects.

**Methods:**

Freshly purified MDC and PDC obtained from HIV+ subjects and healthy controls were cultured in the presence and absence of TLR ligands (poly I∶C or R-848). We evaluated indices of maturation/activation (CD83, CD86, and HLA-DR expression), cytokine secretion (IFN-alpha and IL-6), and ability to activate allogeneic naïve CD4 T-cells to secrete IFN-gamma and IL-2.

**Results:**

MDC from HIV+ subjects had increased spontaneous IL-6 production and increased CD83 and CD86 expression when compared to MDC of controls. MDC IL-6 expression was associated with plasma HIV level. At the same time, poly I∶C induced HLA-DR up-regulation on MDC was reduced in HIV+ persons when compared to controls. The latter finding was associated with impaired ability of MDC from HIV+ subjects to activate allogeneic naïve CD4 T-cells. PDC from HIV+ persons had increased spontaneous and TLR ligand induced IL-6 expression, and increased HLA-DR expression at baseline. The latter was associated with an intact ability of HIV PDC to activate allogeneic naïve CD4 T-cells.

**Conclusion:**

These results have implications for the ability of the HIV+ host to form innate and adaptive responses to HIV and other pathogens.

## Introduction

HIV+ individuals have reduced immune responses to viral antigens and vaccines [1], [2], [3], [4]. HIV disease progression is associated with increased microbial translocation and systemic immune activation [5]. TLR recognition of HIV or microbial products may contribute to systemic immune activation and immune impairment [6], [7]. However, the effects of immune activation on TLR signaling and ability of DC to activate naïve T-cells remain unclear.

Myeloid and Plasmacytoid DC subsets (MDC and PDC) can be defined by expression of specific cell surface markers and TLRs. MDC express BDCA-1 and CD11c, while PDC express BDCA-2, BDCA-4, and CD123. TLR3 is selectively expressed in MDCs, while PDC express TLR7 and 9 [8], [9], [10]. TLR mediated DC activation provides a first line defense against invading pathogens by inducing maturation, cytokine production, and costimulatory molecule expression. Each of these contribute to the generation of pathogen-specific T-cell activation [11], [12].

Alterations in DC numbers, phenotype, and function exist in HIV+ subjects [13], [14], [15], [16], [17], [18]. However, factors underlying these alterations remain poorly understood. Peripheral MDC and PDC numbers are decreased during acute HIV infection [19]. Evidence indicates both cellular redistribution to lymph nodes and cell death may contribute to lower peripheral blood DC numbers [20], [21], [22]. DC TLR responsiveness in HIV infection has been described as both intact [23], [24] and reduced [25], [26]. Conflicting findings may result from differences in patient cohorts, cell culture conditions, and TLR stimulus. Additionally, selective defects in TLR signaling pathways or signaling tolerance may exist in a state of chronic immune activation. For example, LPS tolerance is well described in monocytes, where prior LPS exposure results in impaired LPS induced TLR4 signaling [27]. In addition, during HIV infection Tilton et al described a tolerance effect inhibiting PDC IFN-α production [28]. Whether microbial translocation vs. high HIV levels contribute to TLR signaling tolerance in DC is not clear.

To investigate the relationship between immune activation and DC phenotype and function, we specifically focused here on direct ex vivo analysis of DC activation state, TLR ligand induced DC activation, and TLR ligand dependent, DC dependent naïve CD4 T cell activation. Results show both MDC and PDC from HIV+ subjects are increased in activation phenotype directly *ex vivo*. MDC PDL-1 as well as MDC secreted IL-6 were found to be associated with HIV level. In addition, we found selective defects in MDC and PDC responsiveness to TLR ligand, and MDC ability to activate naive CD4 T-cells. These defects in MDC TLR responsiveness appear related to systemic immune activation.

## Methods

### Study subject identification and IRB approval

HIV+ subjects (n = 31) had detectable HIV antibodies by ELISA and Western Blot, CD4 cell count ≥250, and had not received HIV therapy for at least 3 months. They were seronegative for Hepatitis C and hepatitis B surface antigen negative. The median (range) of clinical indices were as follows: age 41(23–54), plasma HIV 16534(400–490,000) C/ml, CD4 535(115–1139) 10^6^/L, nadir CD4 412(115–999) 10^6^/L, ALT 38(20–71) U/L, AST 22(10–55) U/L, total bilirubin 0.4(0.2–1.0) mg/dL, platelets 225.5(105–485) K/cmm. Eight subjects were known to have had previous therapy for HIV. 45% were female. Healthy control subjects (n = 28) were recruited in a similar age range, 32(21–61), and similar sex distribution (46% female). All study subjects provided written informed consent under the approval of the institutional review board for human studies at University Hospitals of Cleveland.

### Cell isolation

PBMC were fractionated into PDC (positive BDCA-4 selection) and MDC (CD19+ cell depletion followed by BDCA-1 selection) (Miltenyi Biotec). Purity was >80% for MDC (CD11c+ HLA-DR+) and >70% for PDC (BDCA-2+, HLA-DR+), and purity did not differ between study groups.

Naive CD4 T-cells (97% CD3+CD4+CD45RA+cells) from a single healthy leukapheresis donor were negatively selected (Miltenyi Biotec), and frozen in aliquots to be utilized in assays with freshly isolated DC.

### DC phenotype and TLR-induced DC activation

MDC were stained with CD11c-APC, CD83-FITC, HLA-DR-PERCP, and CD86-PE antibodies (BD Biosciences), and PDC were stained with BDCA-2-APC (Miltenyi Biotec), CD83-FITC, HLA-DR-PERCP, and CD86-PE antibodies, and analyzed by flow cytometry (FACSCalibur; BD Biosciences) using CellQuest software (BD Biosciences). Additional phenotypic analysis of MDC was performed using PDL1-PE-CY7 (ebioscience), CD11c-Pacific Blue, PDL2-PE, and ILT3-APC antibodies (BD Bioscience) on a BD LSRII (BD Biosciences).

Isolated PDC and MDC were cultured (20 hr/37°C) in complete RPMI 1640 medium (Invitrogen Life Technologies) supplemented with 1% penicillin-streptomycin,1% L-glutamine, and 5% human AB serum (Gemini Bio-Products) in the presence or absence of TLR ligand. (MDC TLR3 ligand poly I∶C 50 µg/ml, Amersham Biosciences; PDC TLR7 ligand R-848 (resiquimod, 1 µg/ml, InvivoGen). TLR ligands were chosen based on previous experiments comparing DC stimulated with various TLRs for optimal ability to activate allogeneic naïve CD4 T-cells (not shown). Following culture, cells were analyzed by flow cytometric analysis. Cell culture supernatants were analyzed for IL-6 and IFN-α by ELISA (IL-6, eBioscience; IFNα, BioSource International). In addition, IL-6 mRNA level was measured in freshly isolated MDC following RNA isolation with DNase treatment (Qiagen), and cDNA synthesis with RT-PCR analysis (RT^2^ Profiler PCR array SuperArray-ABI StepOnePlus).

### Serum sCD14

Serum sCD14 was measured by ELISA method using a commercial kit, as recommended by the manufactures instruction (Biosource International).

### DC activation of naive CD4 T-cells

Isolated MDC and PDC from each healthy control or HIV infected donor were tested in increasing numbers (1,000, 3,000, and 10,000 cells/well) for ability to activate allogeneic naive CD4 T-cells (2×10^5^/well) that were obtained from one healthy control donor using an IFN- γ ELISPOT assay to measure TLR ligand dependent, DC dependent T cell activation. DC-T-cell co-cultures were performed in the presence or absence of TLR ligand (ligand present for entire culture). Cells were placed into 96-well round-bottom plates (48 hr/37°C), then transferred to ELISPOT plates pre-coated with IFN-γ capture antibody for 20 hr at 37°C. Plates were developed and analyzed as described previously for IFN-γ producing spot forming units [29], [30]. We have previously utilized this assay system that has characteristics of a very low background signal in the absence of DC, and clear signal in the presence of titrated DC numbers [31]. T cell activation is also very sensitive to DC targeted TLR ligand (with no TLR mediated T cell activation in the absence of DC), providing a suitable method for analysis of DC function across a spectrum of allogeneic donors. T-cell IL-2, IL-10, and IL-17 production was analyzed in 3 day supernatants by ELISA (IL-2-ebioscience; IL-10, IL-17A or IL-17F-R&D systems).

Cell viability was evaluated by removing cells following day 3 of culture and staining with AnnexinV-FITC, and CD3-PERCP, CD4-PE, and CD27-APC antibodies (BD Bioscience). Immediate analysis was performed on the BD FACSCaliber.

### Statistical analysis

We used conventional measures of central tendency and dispersion to describe the data and compared continuous variables by Mann-Whitney's *U* test or Kruskal-Wallis' test as needed. We compared proportions by Fisher's exact test. To assess associations between continuous variables, we used Spearman's rank correlation coefficient, as well as partial correlations to control for the effect of intervening variables when needed. All tests of significance are two-sided, and a *p* value<0.05 was considered to be significant.

## Results

### HIV+ subject DC exhibit increased maturation

**Figure 1:**
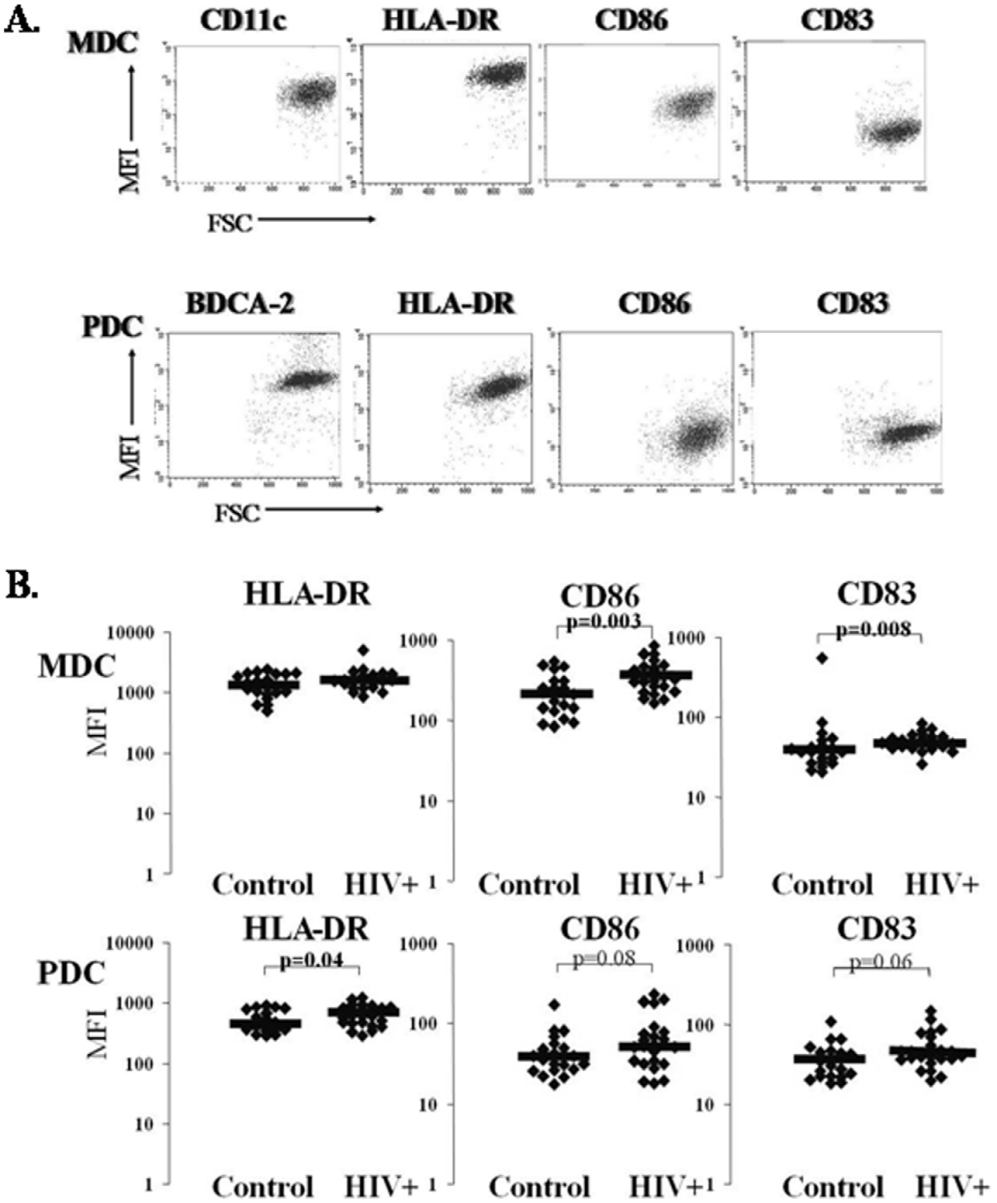
HIV subject DC exhibit increased maturation. **Panel** ***A***. Representative flow cytometric analysis of freshly isolated MDC and PDC from one healthy control. Mean Fluorescent Intensity (**MFI**) for HLA-DR, CD86, and CD83 were analyzed. **Panel** ***B***. Baseline activation/maturation phenotype of freshly isolated MDC and PDC from healthy control (n = 22) and HIV+ subjects (n = 24). The black line represents the median MFI value for each group for HLA-DR, CD86, and CD83.

To identify DC maturation state directly *ex vivo* we evaluated freshly isolated MDC and PDC for maturation marker (CD83, CD86, and HLA-DR) expression as shown in Fig. 1*A*. Comparing DC maturation state between HIV+ subjects and healthy controls, we observed higher CD86 and CD83 expression on HIV+ subject MDC (HIV vs. Controls, 338 vs. 215 CD86 MFI; 50.3 vs. 37.5 CD83 MFI; *p = 0.003* and *p = 0.008 respectively*; Fig. 1*B*). At the same time HIV+ subject PDC exhibited greater HLA-DR expression 614 vs. 469; p = 0.04; Fig. 1*B*) and tended to express more CD86 and CD83 (54 vs. 38 CD86 MFI; and 42 vs. 35 CD83 MFI 42; p = 0.08 and p = 0.06; Fig. 1*B*). These data are consistent with enhanced DC activation during HIV infection, and are in agreement with a previous report [20].

### MDC from HIV+ subjects have less HLA-DR expression in response to poly I∶C stimulation in vitro

**Figure 2:**
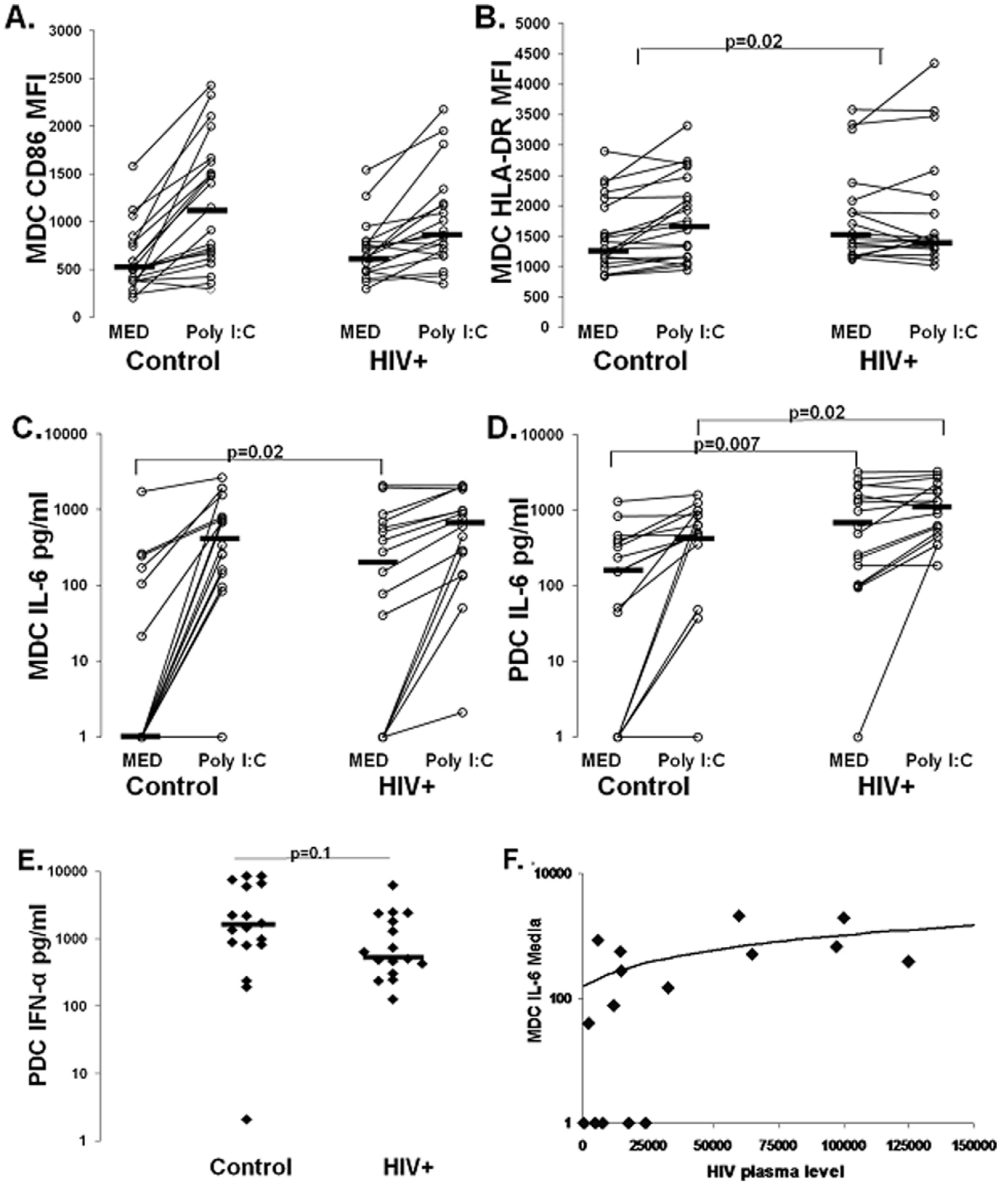
DC TLR ligand responsiveness. **Panels** ***A and B***. CD86 MFI and HLA-DR MFI on isolated MDC following overnight culture in presence of Medium and poly I∶C stimulation in healthy control (n = 21) and HIV+ subjects (n = 18). The p value in panel B is for comparison between the delta HLA-DR MFI for controls and HIV infected subjects . IL-6 production from isolated MDC on a subset of the same subjects (n = 18 control and n = 16 HIV+) (**panel** ***C***) following overnight culture in absence and presence of poly I∶C stimulation. IL-6 production from isolated PDC on a subset of the same subjects (n = 17 control and n = 17 HIV+) (**panel** ***D***) following overnight culture in absence and presence of R848 stimulation. Healthy control (n = 17) and HIV+ subject (n = 16) PDC IFN-α production in response to overnight R848 stimulation is shown in **panel** ***E***. **Panel .** Association between MDC spontaneous IL-6 production and HIV plasma level.

We next evaluated DC subset TLR ligand responsiveness by measuring costimulatory marker (HLA-DR, CD86, and CD83) upregulation and cytokine (IL-6 and IFN-α) secretion following overnight culture in the absence or presence of TLR ligand. As shown in Fig. 2*A*, CD86 expression was upregulated on MDC in most subjects in response to poly I∶C. However, the degree of upregulation of CD86, or delta CD86 MFI, tended to be lower in the HIV+ group (204 vs. 626, p = 0.1, Table 1), with CD86 MFI on MDC of HIV+ subjects tending to be higher following culture in medium and tending to be lower following poly I∶C culture (medium controls 504 vs. HIV 626, p = 0.2; and poly I∶C controls 1031 vs. HIV 856, p = 0.5; Fig. 2*A*). Similar results were observed for HLA-DR expression on MDC, with reduced TLR ligand induced upregulation of HLA-DR on MDC of HIV+ subjects (delta HLA-DR MFI controls 193 vs. HIV −20, p = 0.02, Fig. 2B and Table 1). In contrast, no differences were observed in MDC CD83 expression or upregulation between groups. PDC expression of all markers were comparable between groups in the absence and presence of R848 (not shown), except R848 induced CD86 upregulation tended to be lower in HIV+ subjects (delta CD86 MFI controls 411 vs. HIV 272, p = 0.1, Table 1). These results suggest DC, particularly MDC, from HIV+ subjects have reduced responsiveness to TLR stimulation, as reflected by reduced upregulation of HLA-DR.

**Table 1:** Change in maturation marker expression following in vitro TLR ligand stimulation. Median delta MFI values are given.

		HLA-DR MFI	CD86 MFI	CD83 MFI	IL-6 pg/ml	IFN-α pg/ml
**MDC**	Controls	193	626	11	414	NT
	HIV+	−20	204	7	324	NT
	p value	**p = 0.02**	p = 0.1	p = 0.25	P = 0.22	NT
**PDC**	Controls	407	411	1	282	1515
	HIV+	217	272	2	333	581
	p value	p = 0.5	p = 0.11	p = 0.7	P = 0.8	p = 0.1

To further investigate DC maturation and activation state we evaluated cytokine production. MDC and PDC from HIV+ subjects had increased spontaneous IL-6 production (Fig. 2*C* and 2*D*). This result was further validated using RT-PCR measuring IL-6 mRNA levels in freshly isolated, uncultured MDC from a subset of individuals (n = 4/group). We found a 3 fold increase in IL-6 mRNA levels in MDC from HIV+ subjects compared to controls (p = 0.004, **not shown**). Following TLR stimulation, MDC IL-6 production was comparable between groups, while PDC IL-6 was increased in HIV+ subjects (MDC control 512 pg/ml vs. HIV 676 pg/ml p = 0.4, Fig. 2*C*; and PDC control 495 pg/ml vs. HIV 1020 pg/ml, p = 0.03, Fig. 2*D*). We observed no difference in the degree of IL-6 upregulation following TLR ligand stimulation between groups (MDC controls 414 pg/ml vs. HIV 324 pg/ml, p = 0.22; and PDC controls 282 pg/ml vs. HIV 333 pg/ml, p = 0.8, Table 1). However, we found R848 induced PDC IFN-α production tended to be lower HIV+ subjects (control 1514 pg/ml vs. HIV 581 pg/ml p = 0.1, Fig. 2*E*). Greater spontaneous IL-6 production from the DCs is consistent with prior in vivo activation. Additionally, the intact R848 induced PDC IL-6, yet somewhat reduced IFN-α production, is consistent with selective TLR7 signaling pathway alteration.

Analysis of relationships between immune parameters and clinical indices (HIV level, CD4 T-cell count, sex, age, prior HIV therapy status) indicate a positive correlation between MDC spontaneous IL-6 production and plasma HIV level (r = 0.5, p = 0.02; Fig. 2*E*) consistent with virus level affecting MDC activation state.

### MDC from HIV+ subjects are impaired in activating allogeneic naïve CD4 T-cells, while PDC activating capacity is intact

**Figure 3:**
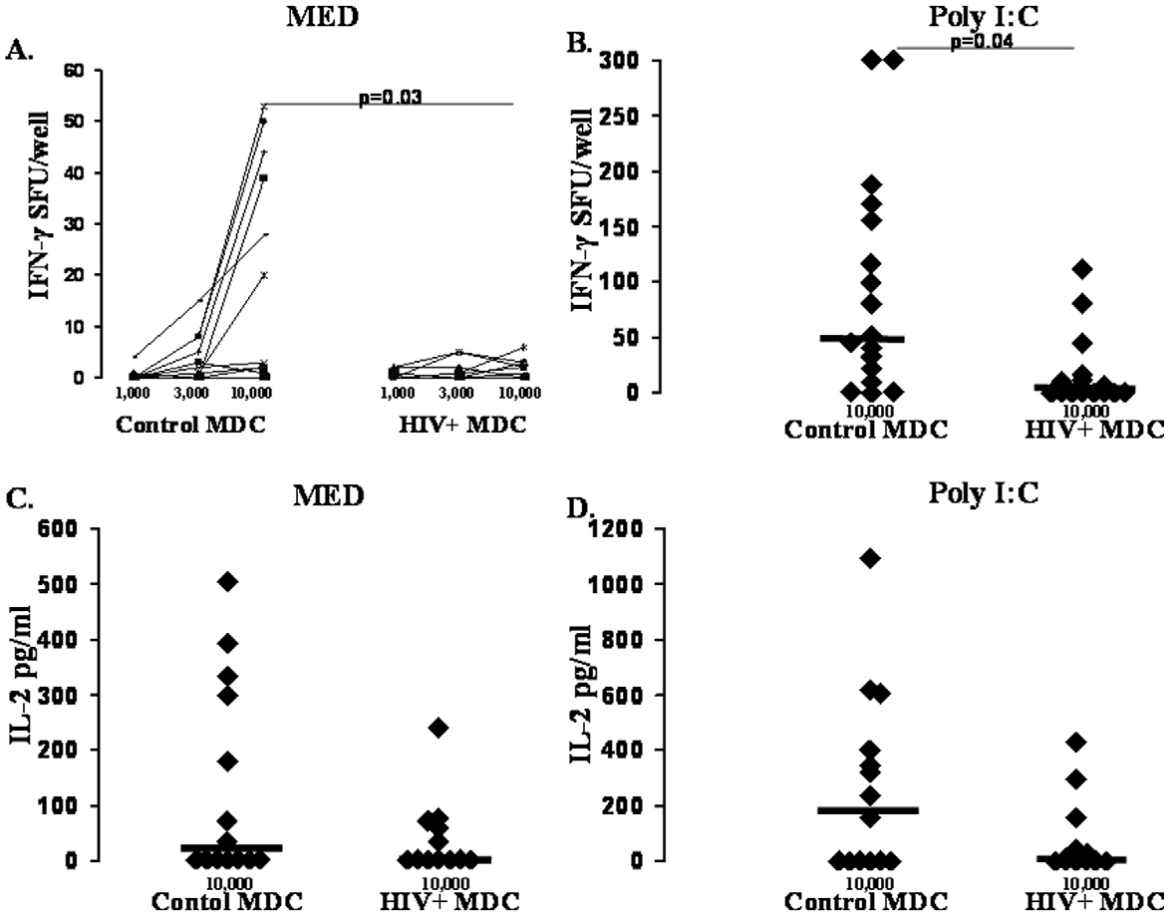
HIV subject MDC are impaired in naïve CD4 T cell activation activity. Freshly prepared MDC from healthy control (n = 17) and HIV+ (n = 18) subjects were used in titrated numbers (*x*-axis) to activate one healthy control subject's allogeneic naive CD4 T-cells to produce IFN-γ or IL-2 (*y*-axis) in a 72-h culture performed in the absence (Medium, **panels** ***A*** **. and** ***C*** **.**), or presence of poly I∶C (**panels** ***B*** **. and** ***D*** **.**). Control cultures of TLR ligand and naïve CD4 T cells resulted in <3 sfu IFN-γ, and data shown are sfu above this background.

**Figure 4:**
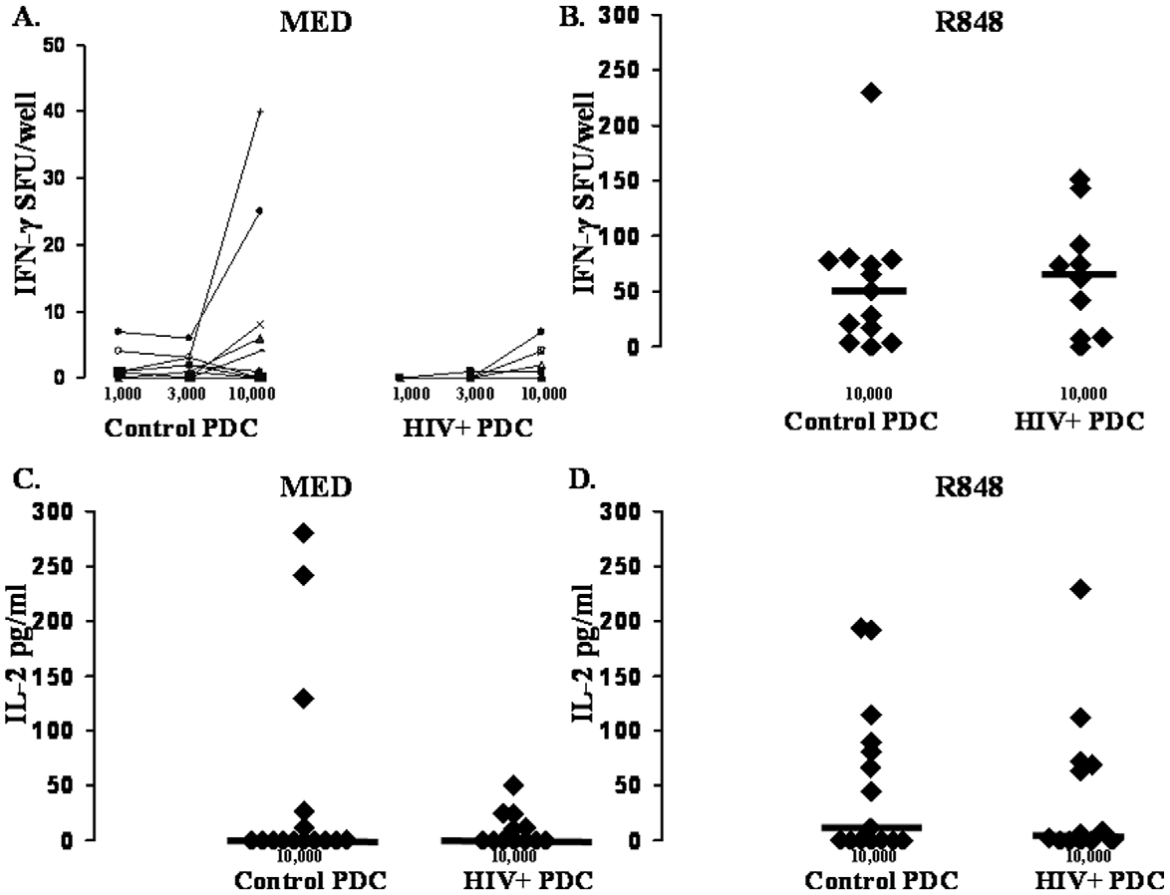
HIV subject PDC are intact for naïve CD4 T cell activation activity. Freshly prepared PDC from healthy control (n = 13) and HIV+ (n = 10) subjects were used in titrated numbers (*x*-axis) to activate one healthy control subject's allogeneic naive CD4 T-cells to produce IFN-γ or IL-2 (*y*-axis) in a 72-h culture performed in the absence (Medium, **panels** ***A*** **. and** ***C*** **.**), or presence of R848 (**panels** ***B*** **. and** ***D*** **.**). Control cultures of TLR ligand and naïve CD4 T cells resulted in <3 sfu IFN-γ, and data shown are sfu above this background.

Prior studies evaluating DC ability to prime T-cell responses in HIV+ subjects have utilized allogeneic PBMC as responder cells and have not focused on the effects of TLR ligands on the ability to enhance activity [18], [32]. Here we specifically focused on TLR ligand dependent DC ability to activate allogeneic naïve CD4 T-cells to produce IFN-γ and IL-2 in the absence and presence of TLR ligand stimulation. MDC from both control and HIV+ subjects induced naïve CD4 T-cell IFN-γ production (Fig. 3*A*). However, HIV+ subject MDC induced significantly fewer IFN-γ producing cells compared to controls (p = 0.03; Fig. 3*A*). In the presence of poly I∶C stimulation, we observed a similar defect in HIV+ subject MDC induction of IFN-γ producing cells (p = 0.04; Fig. 3*B*). Similar results were observed for IL-2 production, though this did not reach statistical significance. HIV+ subject MDC tended to induce less CD4 T-cell IL-2 compared to controls both in the absence and presence of poly I∶C stimulation (0 vs. 16.6 pg/ml, p = 0.4, Fig. 3*C*; and 5.4 vs. 197.7 pg/ml, p = 0.3; Fig. 3*D*). In contrast, the ability of PDC to induce CD4 T-cell IFN-γ and IL-2 was comparable between groups both in the absence and presence of R848 stimulation (Fig. 4). These results indicate a selective defect in the ability of MDC to activate naïve CD4 T-cells in HIV+ subjects.

### PDL1 and PDL 2 expression are increased on MDC from HIV+ subjects and are associated with HIV level

**Figure 5:**
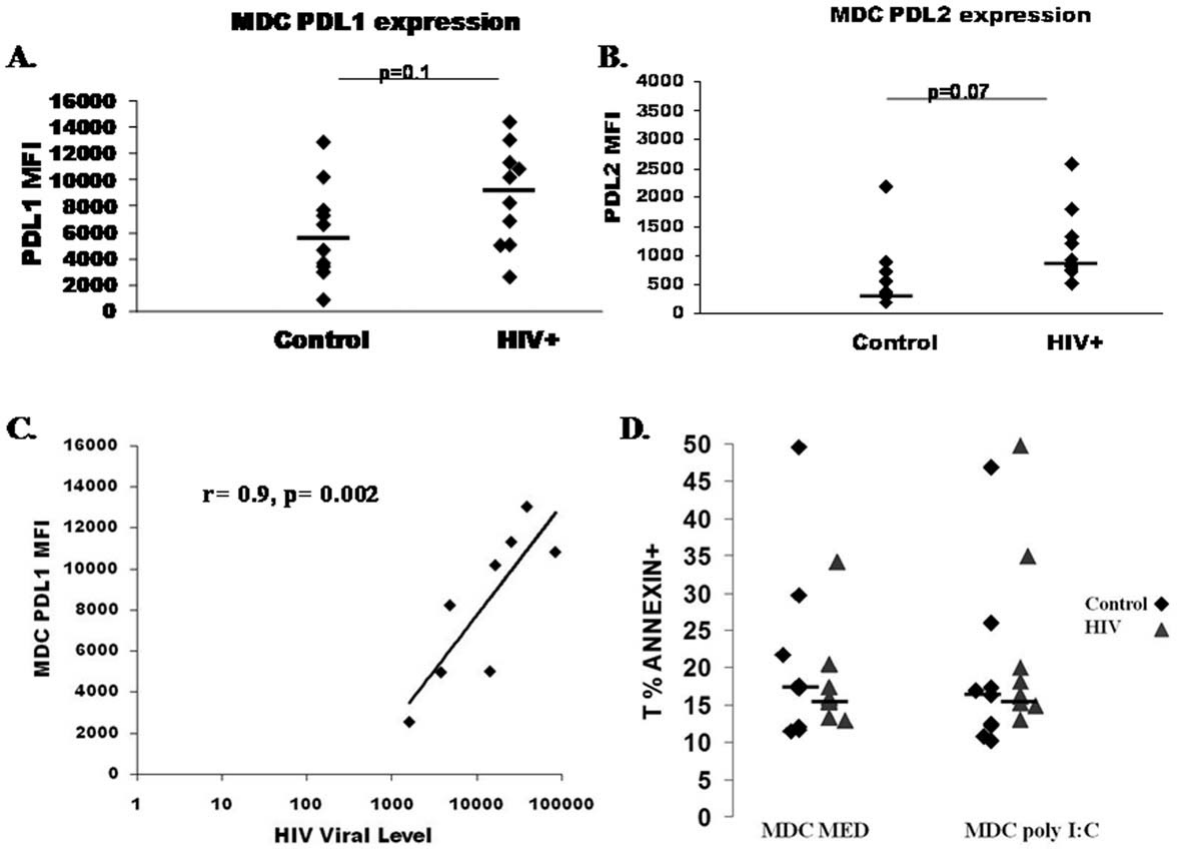
Increased PDL-1 and PDL-2 expression on HIV subject MDC. Isolated MDC were evaluated for expression of inhibitory molecules PDL-1 (**panel** ***A***) and PDL-2 (**panel** ***B***) in control (n = 10) and HIV+ (n = 10) subjects. Black lines represent median MFI value for each group. **Panel** ***C***. Association between PDL-1 MFI on MDC in HIV+ subjects and plasma HIV level. **Panel** ***D***. The percentage of Annexin V+ T-cells are shown following naive T-cell co-cultures with control vs. HIV+ subject MDC (10,000cells/well) in the absence and presence of poly I∶C.

To further investigate the defect in naïve CD4 T-cell activation by HIV+ subject MDC, we measured expression of inhibitory molecules. PD1 is known to inhibit naive T-cell activation [33] and PD1 expression is increased on HIV specific T-cells [34]. The ligand for PD1, PD-L1, has been shown to be expressed at increased levels on MDC, CD14+, and CD19+ cells from HIV+ subjects, and expression is related to markers of HIV disease stage [32], [35] At the same time, TLR ligands derived from HIV directly upregulate PD-L1 on DC and monocytes in vitro [36]. We therefore investigated expression of PD-L1, along with other inhibitory receptors PDL-2 and ILT3 [37], [38], [39]. As shown in Figures 5*A* and 5*B*, PD-L1 and PDL-2 expression tended to be increased on MDC from HIV+ subjects (p = 0.1 and p = 0.07, respectively), while no difference in ILT3 expression was observed between groups (not shown). Furthermore, expression of PD-L1 on MDC was positively correlated with plasma level of HIV RNA (r = 0.9, p = 0.002, Fig. 5*C*) and negatively correlated with CD4 T-cell count (r = −0.76, p = 0.03, not shown).

To determine whether the increased PDL-1 or PDL-2 expression on MDC from HIV+ subjects contributed to T-cell apoptosis [40], we measured AnnexinV staining on T-cells following 3 day naive T-cell-MDC cocultures. We did not observe any difference in percentage of AnnexinV+ T-cells comparing naive T-cells cocultured with MDC from HIV+ subjects vs. controls in either the absence or presence of TLR ligand (p = 0.7 and p = 0.3; Fig. 5*D*). We also evaluated whether MDC from HIV+ subjects were more likely to induce T-cell IL-10 or IL-17 secretion. We observed limited induction of IL-10 in both the absence and presence of TLR stimulation in control (3.5 pg/ml and 5.3 pg/ml) and HIV+ subject (2 pg/ml and 2.6 pg/ml) MDC-T-cell cocultures with no statistical difference between groups (not shown). IL-17 levels were undetectable by ELISA method (not shown). We therefore found no evidence of CD4 cytokine variation in response to HIV+ subject MDC.

### Reduced HLA-DR and CD86 upregulation on MDC of HIV+ subjects following TLR ligand stimulation are associated with reduced naïve CD4 T-cell activation

To investigate the mechanism underlying the failure of MDC from HIV+ subjects to activate naïve CD4 T-cells, we evaluated associations between MDC activation state and naïve CD4 T-cell response. Within the HIV+ group, MDC responsiveness to TLR ligand, as measured by HLA-DR, CD86, and IL-6 upregulation, was correlated with ability to activate naïve CD4 T-cells (r = 0.47, p = 0.08; r = 0.6, p = 0.03; and r = 0.55, p = 0.03, respectively). In control subjects, baseline HLA-DR expression and upregulation of CD86 and IL-6 in response to TLR ligand were correlated with MDC ability to active naive CD4 T-cells (r = 0.6, p = 0.02; r = 0.6, p = 0.01; and r = 0.7, p = 0.01). These data are consistent with HLA-DR and CD86 playing an important role in naïve T-cell activation in both control and HIV+ subject sample cultures, and are consistent with our earlier data indicating a strong costimulatory dependence in this naïve CD4 T-cell activation assay system [31]. These data suggest that the reduced upregulation of costimulatory molecules in MDC of HIV+ subjects following TLR ligand stimulation may contribute to the observed defect in ability to activate naïve CD4 T-cells.

Biomarkers of systemic immune activation and microbial translocation have been associated with increased activation state of CD8 T-cells and monocytes in HIV+ individuals [5], so we investigated the relation between immune activation and DC phenotype and function. We measured sCD14, a marker of systemic immune activation that is shed from monocytes upon LPS binding and activation [41]. Consistent with prior literature [5],[42], we observed greater sCD14 in the serum of HIV+ subjects compared to controls (2.35 µg/ml vs. 1.6 µg/ml p = 0.03; not shown). Interestingly, we found a negative correlation between sCD14 level and MDC HLA-DR expression observed after TLR ligand stimulation (r = −0.9, p = 0.04; not shown) in HIV+ subjects, while no such relationship was observed in healthy control samples.

## Discussion

We evaluated DC subset phenotype and function in relation to systemic immune activation during HIV disease. Results indicate both MDC and PDC from HIV+ subjects have increased expression of maturation markers when analyzed directly *ex vivo*. In addition, we observed MDC from HIV+ subjects to be refractory in response to TLR stimulation, with reduced upregulation of HLA-DR expression in response to poly I∶C. PDC from HIV+ subjects tended to have reduced upregulation of both CD86 expression and IFN-α production following TLR7 stimulation. Further investigation revealed MDC dependent naive CD4 T-cell activation was impaired in HIV+ subjects, while PDC dependent naive CD4 T-cell activation was intact. Results indicate multiple factors that may contribute to the MDC defect, including increased inhibitory molecule expression (PDL-1 and PDL-2) which is associated with HIV plasma level, and defective responsiveness to TLR ligand stimulation which is associated with systemic immune activation (serum sCD14). Circulating immature DC are thought to exist in a quiescent state, expressing low levels of costimulatory molecules and cytokines [43]. Upon recognition of microbial pathogens, DC mature, express costimulatory molecules, and secrete cytokines to facilitate T-cell activation [44], [45]. Our results indicate circulating MDC and PDC from HIV+ subjects express increased levels of costimulatory molecules and IL-6 directly *ex vivo*, suggesting DC maturation is perturbed during HIV disease. These phenotypic data are supported by a previous report by Dillon et al., in which both DC subsets were found to have altered chemokine receptor and maturation marker expression [20]. A new finding here is the observation of a positive correlation between spontaneous MDC IL-6 expression and plasma HIV levels, consistent with the relationship between DC CD40 expression and HIV RNA noted by Dillon et al. These results suggest HIV may directly perturb DC maturation phenotype in vivo, possibly through DC interactions with HIV proteins and/or viral RNA, both shown to induce DC maturation in vitro [6], [46]. Additionally, monocyte activation through direct contact with translocated microbial products may indirectly affect DC activation/maturation state, and our observed association between serum sCD14 and DC HLA-DR expression in response to TLR ligand could be consistent with the latter.

The ability of immature DC to mature, via TLR activation, is a critical step in generating an effective immune response. In our HIV+ subject samples, we observed selective defects in MDC and PDC responsiveness to TLR stimulation. One previous study evaluating TLR responsiveness in HIV+ subjects reported TLR7 stimulated PBMC have decreased PDC CD83 expression and IFN-α production [25], [28], while another study reported increased TNF-α production [23]. Discrepancies in TLR responsiveness are likely due to differences in the TLR ligand used and response parameters measured. In fact, our data support this notion in that alterations in DC TLR signaling pathways are selective and discrete. Specifically, ability of PDC to secrete IFN-α and upregulate CD86 in response to TLR7 stimulation appears impaired, while IL-6 production is intact. At the same time, MDC have reduced HLA-DR upregulation in response to TLR3 stimulation, despite having intact IL-6 production. One possible mechanism by which MDC and PDC become partially attenuated in response to TLR stimulation is through prior activation in vivo with resulting refractory responsiveness, as previously discussed in the context of PDC IFN-α production [28]. This concept is supported here by the baseline increase in DC activation state.

Alterations in DC dependant activation of T-cells have been described in the setting of chronic HIV infection, using purified DC subsets [18], [47]. Specifically, MDC and PDC from HIV+ subjects were found to be impaired in stimulating unfractionated PBMC T-cell proliferation and cytokine production. The mechanisms behind these observations were not fully understood [18], and these assays likely reflect a combination of memory and naive T cell activation. Since TLR responsiveness may be particularly important for DC activation and ability of DC to activate naïve T-cells, we focused here specifically on this interaction. We also focused on naïve T cell activation, eliminating any contribution of memory T cell activation. We found MDC from HIV+ subjects, despite having increased cytokine and costimulatory molecule expression at baseline, are less able to activate naïve CD4 T-cells both in the absence and presence of TLR3 stimulation. Observed correlations between ability to upregulate costimulatory molecules CD86 and HLA-DR in response to TLR stimulation, and ability to activate naïve CD4 T-cells, suggest a common mechanism underlies decreased responsiveness to TLR stimulation and defective MDC dependant naïve T-cell activation. Additionally, increased PDL1 and PDL2 expression on HIV+ subject MDC may contribute to impaired naïve CD4 T-cell activation as has been previously suggested [48]. Our data are in support of this possibility, and this is supported further by previous data showing enhanced MDC dependant memory T-cell activation in the presence of PDL1-PD1 blockade [32]. Notably, we did not find an alteration in T cell annexin staining in the present assays, suggesting the PDL1/2 effect does not likely result in T cell death during the time frame of the assay performed here. In contrast to MDC, PDC were found here to have intact naïve T-cell activation activity, and this was positively correlated with TLR7 induced HLA-DR expression and IL-6 production, which were also intact in HIV+ subjects. These results indicate a distinct functional defect in MDC naive T-cell activation, emphasizing selective alterations exist in MDC and PDC during HIV infection.

Engagement of different signaling pathways in separate DC subsets may contribute to the selective defects observed. One possibility is that differential activation of DC subsets occurs through different pathogen recognition receptors by either HIV, or other microbial products present during HIV infection. These stimuli could engage distinct signaling cascades within each DC subset, rendering each cell type with selective defects in responsiveness to subsequent TLR stimulation. Indeed, HIV ssRNA has been shown to directly induce MYD88 dependent DC maturation [6]. Here we observe a direct correlation between HIV level and MDC PDL1/2 expression and IL-6 secretion, suggesting HIV may play a direct role in activating DC in vivo. Of course, indirect effects remain a possibility as well. Additionally, we also observed a correlation between reduced MDC upregulation of HLA-DR expression following TLR stimulation and increased serum sCD14. sCD14 is thought to be released from cells activated by LPS [41], suggesting that systemic exposure to translocated microbial products in HIV infection [5] may also contribute to the observed DC defect. Though the consequences of altered DC maturation state are not fully understood, increased cytokine production and refractory responsiveness are likely detrimental to development of an effective immune response. For instance, increased serum IL-6 level has been predictive for risk of cardiovascular disease in the general population, and mortality in HIV+ persons [49], [50]. Therefore, excess IL-6 production by DC in HIV+ subjects may contribute to morbidity. Future studies aimed at distinguishing the direct effects of HIV verses those of other microbial elements indirectly resulting from HIV infection on immune cell functions are required to guide more effective vaccine strategies and immuno-modulatory therapies in the setting of HIV infection.
